# *Galleria mellonella* and the Bac-to-Bac Expression System: A Convenient Model for Testing Molecules Intended to Genetically Enhance Baculovirus Pathogenicity

**DOI:** 10.3390/insects16090923

**Published:** 2025-09-02

**Authors:** Sergey A. Timofeev, Anastasia G. Shukhalova, Alsu M. Utkuzova, Ruslan R. Fadeev, Viacheslav V. Dolgikh, Igor V. Senderskiy

**Affiliations:** All-Russian Institute of Plant Protection, Podbelskogo 3, 196608 St. Petersburg, Russia; nastyadzh@mail.ru (A.G.S.); alsuvizr@mail.ru (A.M.U.); fadeeff.rusln@gmail.com (R.R.F.); dol1slav@yahoo.com (V.V.D.); senderskiy@mail.ru (I.V.S.)

**Keywords:** baculoviruses, biopesticides, genetic modification, *Galleria mellonella*

## Abstract

The use of viruses as natural pathogens of pest insects is a promising approach in biocontrol. The main drawback of this approach is its relatively slow mode of action, which could be addressed through genetic modifications of the strains used. In this article, we propose the use of the commercially available Bac-to-Bac system for infecting pest insects with a recombinant virus and assessing the impact of various molecules on their viability. Using the laboratory-friendly model organism *Galleria mellonella* and baculovirus carrying the *eGFP* gene, we demonstrated a 100% infection rate, with a consistent and stable spread of the viruses throughout insect bodies and a gradual increase in recombinant protein expression. Notably, at day 5 post-infection the insects remained viable and active. This approach enables the identification of pathogenic effects caused by the virus-induced expression of other molecules, such as toxins.

## Simple Summary

Natural insect pathogens, such as viruses, can be used for the biological control of crop pests. Genetic modification of these viral strains can significantly enhance the effectiveness of this approach. In recent years, numerous molecules with potential for such modifications have been described; for example, toxins from the venoms of predatory and parasitic insects. However, only a small fraction of these molecules have been studied in the context of genetic engineering. In this article, we propose a convenient and safe model system of testing such molecules based on a commercially available viral strain from the Bac-to-Bac protein expression system and the laboratory-friendly model insect *Galleria mellonella*. We demonstrate high stability of experimental infection in insects, and consistent expression of the foreign eGFP protein introduced via virus modification. We also show that the virus exhibits relatively low pathogenicity in our system, making it a suitable tool for evaluating the enhanced virulence achieved through the use of various toxins and similar bioactive molecules in future genetic modifications.

## 1. Introduction

The use of natural pathogens of pest insects, such as fungi, bacteria, and viruses, is a promising approach in biological crop protection due to its relative safety for the environment and human health compared to the use of synthetic pesticides [1,2]. In the case of viruses, members of the Baculoviridae family, commonly known as baculoviruses, are typically employed against lepidopteran pests [3]. Their high specificity to particular insect species, along with their inability to infect vertebrates, makes them a safe and convenient tool. The main drawback of this approach is the relatively slow action of the viruses: several days or even weeks may pass from the moment of infection to the death of the insect or the cessation of its feeding [4]. One promising strategy to overcome this limitation is the development of recombinant virus strains that force infected insect cells to produce molecules with a detrimental effect on the host’s viability, such as toxins [5,6,7].

To date, dozens of recombinant strains of various baculovirus species have been created and tested on lepidopteran pests. However, a significant proportion of these strains have been modified using the same well-characterized toxin sequences that have already demonstrated effectiveness [7]. Examples include TxP-I from the venom of the predatory mite *Pyemotes tritici*, and AaIT from the venom of the scorpion *Androctonus australis* [7,8,9,10]. The use of a broader variety of molecules for the development and testing of recombinant viruses is limited by the highly labor-intensive process of generating such strains. This approach typically involves infecting insect cell cultures co-transfected with a plasmid carrying the gene of interest, followed by selection of recombinant viruses in which homologous recombination has occurred [5]. Unfortunately, this method is of inherently low efficiency. Moreover, in many cases, toxins that have proven effective when directly injected into an insect’s body cavity fail to exhibit activity when synthesized by the virus-infected insect’s own cells, and such recombinant strains do not demonstrate enhanced pathogenicity compared to the wild-type virus [5,6,7]. Finally, another challenging task is to detect the specific effects of genetic modifications against the background of the virus’s inherent pathogenicity. Most researchers, quite logically, aim at enhancement of the already potent pathogenicity of viral strains through genetic modification. As a result, demonstrating statistically significant differences between wild-type and modified strains often requires infecting very large cohorts of insects to increase the power of statistical analysis [5].

Thus, to simplify the testing of new molecules for the development of recombinant baculoviruses with enhanced virulence, it is necessary to streamline both the creation of recombinant strains and the selection of a model virus-host system in which infection and heterologous protein expression occur reliably while the pathogen itself exhibits relatively low virulence, making it easier to detect the effects of genetic modification. To address the first challenge, a method was developed as early as the 20th century: transposon-mediated insertion of foreign genes into a baculovirus genome propagated in *Escherichia coli* [11]. The modified *Autographa californica* multicapsid nucleopolyhedrovirus (*Ac*MNPV) strain and the recombination method proposed by the authors whereby the viral genome is modified within bacterial cells are still used today in commercial systems for recombinant protein production in insect cell cultures, such as Bac-to-Bac™.

Although this method is not suitable for the development of baculovirus-based biopesticides because the modified strain was specifically designed for protein expression and is incapable of forming the environmentally stable viral occlusion stage (polyhedra) [11], when studying toxins from the venom of the parasitoid wasp *Habrobracon hebetor*, we discussed the potential of this system for testing new molecules intended for the development of recombinant viruses as biopesticides. Analysis of insect cell cultures infected with recombinant virus allowed us to determine whether the inserted molecule was synthesized in a soluble form and whether it was secreted from the transfected cells [12]. In the present study, we sought to go a step further by using this system in a non-standard way: direct infection of live pest insects (and not cultured cells) with the recombinant virus. Despite the absence of an oral infection stage in these viruses, we hypothesized that viral particles produced in cell culture could still be suitable for infecting insects via direct injection into the hemolymph. Therefore, it was necessary to select a lepidopteran species that could be efficiently infected through this method, a species whose cells would support robust production of recombinant proteins while the overall course of infection remained relatively mild, thus facilitating the distinction between the effects of the tested proteins and those caused by the virus itself.

For the purposes of this study, we selected the lepidopteran pest *Galleria mellonella*, whose larvae can be easily reared year-round under laboratory conditions [13]. Grown-up larvae reach a sufficiently large size to allow for convenient injections without the need for specialized equipment such as micromanipulators. Meanwhile, their biological characteristics facilitate the maintenance of large numbers of individuals in experimental settings. In addition, the possibility of infection of this pest species with *Ac*MNPV has been shown previously [14]. For convenient visualization of the infection process and recombinant protein expression, we used a viral strain carrying a sequence encoding enhanced green fluorescent protein (eGFP) for infection.

## 2. Materials and Methods

The culture of *G. melonella* and its maintenance conditions were described earlier [15]. Recombinant *Ac*MNPV strain from Bac-to-Bac™ expression system carrying *eGFP* sequence was obtained previously [16]. Baculovirus stock was obtained after five passages in the *Spodoptera frugiperda* Sf9 cell line, following the same procedure as for preparing a baculovirus stock for protein expression in cell culture, according to the Bac-to-Bac™ manufacturer’s protocol (Thermo Fisher Scientific, Waltham, MA, USA). For infection of insects, a culture medium with virus titer of 1 × 10^5^ plaque forming units (pfu)/mL was frozen at −80 °C. Preservation of the same infectivity after defrosting was carried out according to the manufacturer’s protocol for Bac-to-Bac™. *Galleria mellonella* larvae at the fifth and sixth instar stages were infected by injecting 10 µL of thawed viral stock using a Hamilton injector. Control larvae were injected with the same volume of virus-free culture medium. The insects were kept in glass Petri dishes in groups of 5–10 individuals, fed with artificial diet, at room temperature. For microscopic observation, an Axio Imager M1 fluorescent microscope (Carl Zeiss, Oberkochen, Germany) was used.

For molecular analysis, the larvae were frozen at −80 °C. To prepare protein samples for immunoblotting, a tissue fragment of approximately 50 mg was chipped off from the frozen larvae and homogenized in 300 µL of sample buffer (65 mM Tris-HCl, pH 6.8, 2% SDS, 5% 2-mercaptoethanol, 10% glycerol) in 1.5 mL microcentrifuge tubes using homogenizers from the Lumepure kit (Lumiprobe, MD, USA). SDS-PAGE and immunoblotting were performed as previously described [12], using anti-eGFP antibodies (Evrogen, Moscow, Russia) at a 1:3000 dilution. For the extraction of total RNA, the same fragment of frozen caterpillars was homogenized with a mortar and pestle in liquid nitrogen, and MagZol Reagent (Magen, Guangzhou, China) and deoxyribonuclease I (Evrogen, Moscow, Russia) were used according to the manufacturer’s instructions. The cDNA synthesis was performed using MMLV reverse transcriptase and oligo-dT18 primers (Evrogen, Moscow, Russia) with 1.5 µg of total RNA as a template. RT-PCR and Real-time qPCR were performed as previously described [17] using primers to *eGFP* (CCATGAATTCATGGTGAGCAAGGGCGAGGAGCTG forward and GTCACTCGAGTTACTTGTACAGCTCGTCCATGCCGA reverse) and *G. melonella* actin fragment (gene bank accession number XM_026904349.3, ATCGGCAATGAGCGGTTCC forward and GGGCCAAGGCGGTGATTTC reverse).

## 3. Results

In total, we conducted four rounds of experimental infection of G. mellonella larvae. In the first experiment, fifteen larvae were injected with the eGFP-expressing virus, and five larvae received virus-free culture medium as controls. On the 1st, 3rd, and 5th days post-injection (dpi), three infected larvae and one control larva were examined microscopically. The remaining three independent infections were performed using the same protocol: 20 experimental and 10 control larvae were used in each trial. For each of these experiments, one infected and one control larva were examined microscopically on days 1, 3, 5, and 7, and three infected and one control larva were frozen on each of those days for molecular analysis. The remaining larvae from all experiments were monitored until day 10 post-injection.

**Figure 1:**
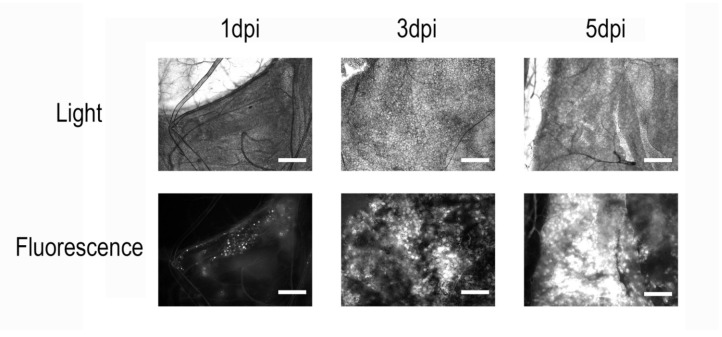
Microscopy of *Galleria mellonella* fat body tissue after infection with a recombinant *Ac*MNPV strain carrying an eGFP-encoding sequence. Scale bar = 100 µm. On the first day post-infection, only a few localized regions with fluorescent signal can be observed. By day 3, widespread infection becomes apparent, and by day 5, nearly all insect tissues are infected, with visible accumulation of the recombinant protein in cells.

In all microscopically examined larvae across the four independent infections, we observed a consistent pattern (Figure 1). On the first day post-infection, only a few small and isolated fluorescent areas could be detected in the fat body tissue, indicating the early stage of infection and the onset of recombinant protein synthesis. At 3 dpi, distinct but already-extensive zones of infection and eGFP accumulation were visible in the fat body cells. At 5 dpi, the entire fat body tissue of infected larvae was visibly infected. Moreover, the strong fluorescence of all tissues, including the hemolymph, became clearly visible to the naked eye on the microscope slide table. Interestingly, infected larvae also developed a distinct greenish hue compared to controls, even in the absence of fluorescence excitation. No fluorescence was detected in control insects.

**Figure 2:**
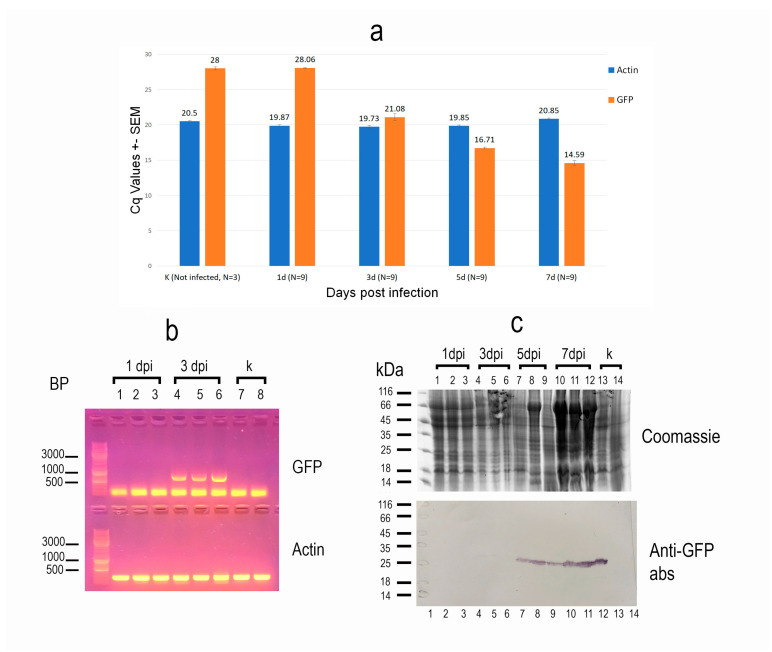
Characteristics of recombinant protein expression in *Galleria mellonella* caterpillars following infection with a recombinant *Ac*MNPV strain carrying an eGFP-encoding sequence. (**a**) Real-time qPCR analysis of *eGFP* expression. Insect actin was used as the reference gene. N—number of insects analyzed per sample. Expression of the recombinant protein was detected starting at 3 days post-infection (dpi), with a steady increase observed at days 5 and 7. Low standard error values indicate consistent expression levels across individuals on each respective day post-infection. (**b**) Reverse transcription PCR (RT-PCR) analysis of eGFP expression at early time points post-infection. Insect actin (250 bp fragment) served as the reference gene. Lanes 1–3: three individuals at 1 dpi; lanes 4–6: 3 dpi; lanes 7–8: uninfected controls. *eGFP* expression was detectable at 3 dpi but not at 1 dpi. (**c**) Immunoblot analysis of recombinant protein accumulation in insect tissues from different individuals using anti-eGFP antibodies. Lanes 1–3: 1 dpi; lanes 4–6: 3 dpi; lanes 7–9: 5 dpi; lanes 10–12: 7 dpi; lanes 13–14: uninfected controls. Accumulation of the recombinant protein was minimal at 3 dpi but became clearly detectable by 5 dpi.

To assess the dynamics of recombinant protein expression in insects, we analyzed three infected larvae from each of three independent infections at 1, 3, 5, and 7 dpi, as well as one control larva per experiment, using real-time PCR. The *G. mellonella* actin gene, which is constitutively expressed, was used as a reference. Interestingly, although microscopic examination revealed the first signs of infection at 1 dpi, the observed Cq values for eGFP expression were indistinguishable from those of the controls, indicating that this method was not sensitive enough to detect infection at such an early stage (Figure 2a). However, starting from day 3, a steady increase in recombinant protein expression level was observed. Moreover, the expression levels were consistent across individuals from different experiments at the same time points post-infection.

To confirm that infection at 1 dpi cannot be reliably detected by PCR, and to demonstrate that the observed Cq values corresponded specifically to *eGFP* amplification rather than a nonspecific product, samples from three larvae at 1 and 3 dpi, as well as from two control larvae, were additionally analyzed using conventional RT-PCR. The number of amplification cycles was increased to 40, compared to the standard protocol. As expected, under these conditions, a product matching the expected size of the *eGFP* sequence was detected only in the 3 dpi samples, but not in the 1 dpi or control samples (Figure 2b).

Finally, three samples of larvae from each time point of the last infection experiment, as well as two uninfected controls, were analyzed not only for synthesis of recombinant protein, but also its accumulation in insect tissues, using immunoblotting with anti-eGFP antibodies. The results confirmed that the recombinant product was not detectable before 3 dpi, when barely detectable amounts were recorded. Significant accumulation of the virus-delivered protein in the tissues was only observed starting at 5 dpi (Figure 2c).

Out of all the seventy-five infected and thirty-five control larvae, three infected and two control individuals died spontaneously on day 1 post-injection, and only two additional infected individuals died between days 7 and 10 of the experiment. We assume that the early deaths were due to injection-related trauma rather than the infection itself. At 5 dpi, all infected larvae were actively moving and feeding. A noticeable impact on their activity was observed only at 7 dpi, when the larvae still responded to mechanical stimulation but ceased to locomote and feed. All 13 infected larvae that survived until day 10 (and were not used for sample preparation) continued to respond to mechanical stimuli, although microscopic analysis revealed large-scale lytic processes in their tissues).

## 4. Discussion

The utilization of genetically modified organisms in plant protection is currently predominantly confined to the development of pathogen-resistant crops [18]. There are as yet no commercially available insecticides derived from genetically modified viruses. This is primarily due to two main factors. Firstly, there has been a paucity of field trials demonstrating the safety of such insecticides. This is particularly applicable to recombinant viruses, wherein there exists a theoretical risk of gene transmission to closely related virus species, posing a potential threat to non-target insect species [5,6]. Secondly, and notably significant in our view, is societal resistance to genetically modified organisms [5,6,7,19]. Nevertheless, researchers observe an escalating tolerance toward genetically modified organisms in numerous countries, and anticipate a widespread introduction of such insecticides to the market within the next 20–30 years [19,20]. Consequently, the accumulation of knowledge and the development of experimental insecticides based on genetically modified organisms in the laboratory will provide a robust knowledge base for the future creation of commercial insecticides. Even among the molecules with potential for use in viral genetic modification and the development of new biopesticides that have been identified in the past decade or earlier, only a few have been studied in this context [7,21,22,23]. In this article, we have attempted to propose a convenient model system for evaluating the potential of such molecules. This approach allows for the selection of the most effective candidates with minimal effort, and it could be used in the future to develop new biopesticides once their practical application becomes feasible. Although the baculoviruses obtained in this system cannot be directly used as biopesticides due to the absence of an oral infection stage, we emphasize the value of our system specifically for convenient testing of a wide range of different molecules. Screening a large number of candidates with a simplified system and then applying the most promising ones to the actual development of viral biopesticides seems to us a more efficient approach than creating fully functional prototypes for each molecule from the start, which would require considerably greater effort each time.

Summarizing the obtained experimental data, we can conclude that injection of a fixed dose of an *Ac*MNPV strain from the Bac-to-Bac™ expression system carrying the *eGFP* sequence into the hemolymph of *G. mellonella* larvae led to infection of all injected individuals. The rate of infection spread, the level of recombinant protein expression, and the accumulation rate of the recombinant product in the host cells were highly consistent across the insect specimens. Specifically in the case of GFP, whose fluorescence can be detected microscopically even at extremely low concentrations, we were able to detect the recombinant protein as early as 1 dpi; however, in all cases, we were able to assume that between 3 and 7 dpi, the expression level of the recombinant protein became very high, and detectable with any of the methods used. In addition, noticeable effects on larval viability were observed only at the end of this period.

Based upon these observations, we conclude that this model system is well suited for testing new molecules that may enhance the virulence of genetically modified baculovirus strains targeting insect pests. The use of a convenient commercial system enables the easy generation of a large number of recombinant virus strains carrying various inserted molecules and modifications, such as different signal secretory peptides in the case of protein insertion. The consistent infection dynamics and expression levels, along with the low virulence of the employed viral strain in *G. mellonella*, allow for the use of smaller insect sample sizes when assessing the effect of specific molecular insertions, compared to infections with unmodified viruses. An important feature of our testing model is its safety: due to the absence of an oral transmission stage in the strain used, infection experiments can be conducted without the need to isolate the experimental insects from others in the laboratory. Molecules identified as promising through this model may subsequently be used for modifying other baculoviruses that have potential for application as biopesticides.

## Data Availability

The raw data used for creation of schemes in this article is presented in Table S1 in Supplementary Materials.

## Supplementary Materials

The following supporting information can be downloaded at: https://www.mdpi.com/article/10.3390/insects16090923/s1, Table S1: Real-time qPCR results on the basis of which Figure 2 is constructed.

## Abbreviations

The following abbreviations are used in this manuscript:

eGFP	Enhanced green fluorescent protein
*Ac*MNPV	*Autographa californica* multicapsid nucleopolyhedrovirus
SDS	Sodium dodecyl sulfate
RT-qPCR	Reverse transcription quantitative polymerase chain reaction
DPI	Day post-infection
Cq	Quantification cycle
